# Contrasting effects of sunitinib within in vivo models of metastasis

**DOI:** 10.1007/s10456-012-9291-z

**Published:** 2012-07-28

**Authors:** Jonathan C. Welti, Thomas Powles, Shane Foo, Morgane Gourlaouen, Natasha Preece, Julie Foster, Sophia Frentzas, Demelza Bird, Kevin Sharpe, Antoinette van Weverwijk, David Robertson, Julie Soffe, Janine T. Erler, Roberto Pili, Caroline J. Springer, Stephen J. Mather, Andrew R. Reynolds

**Affiliations:** 1 Tumour Biology Team, Breakthrough Breast Cancer Research Centre, The Institute of Cancer Research, Fulham Road, London, SW3 6JB UK; 2 Centre for Molecular Oncology, Barts Cancer Institute—A CR-UK Centre of Excellence, Queen Mary University of London, John Vane Science Centre, Charterhouse Square, London, EC1M 6BQ UK; 3 Gene and Oncogene Targeting Team, The Institute of Cancer Research, Cancer Research UK Centre for Cancer Therapeutics, 15 Cotswold Road, Sutton, Surrey, SM2 5NG UK; 4 Tumour Hypoxia and Metastasis Team, The Institute of Cancer Research, Fulham Road, London, SW3 6JB UK; 5 Molecular Cell Biology Team, Breakthrough Breast Cancer Research Centre, The Institute of Cancer Research, Fulham Road, London, SW3 6JB UK; 6 Roswell Park Cancer Institute, Elm & Carlton Streets, Buffalo, NY 14263-0001 USA

**Keywords:** Angiogenesis, Metastasis, Resistance, VEGF, Breast, Renal

## Abstract

Sunitinib is a potent and clinically approved tyrosine kinase inhibitor that can suppress tumour growth by inhibiting angiogenesis. However, conflicting data exist regarding the effects of this drug on the growth of metastases in preclinical models. Here we use 4T1 and RENCA tumour cells, which both form lung metastases in Balb/c mice, to re-address the effects of sunitinib on the progression of metastatic disease in mice. We show that treatment of mice with sunitinib prior to intravenous injection of tumour cells can promote the seeding and growth of 4T1 lung metastases, but not RENCA lung metastases, showing that this effect is cell line dependent. However, increased metastasis occurred only upon administration of a very high sunitinib dose, but not when lower, clinically relevant doses were used. Mechanistically, high dose sunitinib led to a pericyte depletion effect in the lung vasculature that correlated with increased seeding of metastasis. By administering sunitinib to mice after intravenous injection of tumour cells, we demonstrate that while sunitinib does not inhibit the growth of 4T1 lung tumour nodules, it does block the growth of RENCA lung tumour nodules. This contrasting response was correlated with increased myeloid cell recruitment and persistent vascularisation in 4T1 tumours, whereas RENCA tumours recruited less myeloid cells and were more profoundly devascularised upon sunitinib treatment. Finally, we show that progression of 4T1 tumours in sunitinib treated mice results in increased hypoxia and increased glucose metabolism in these tumours and that this is associated with a poor outcome. Taken together, these data suggest that the effects of sunitinib on tumour progression are dose-dependent and tumour model-dependent. These findings have relevance for understanding how anti-angiogenic agents may influence disease progression when used in the adjuvant or metastatic setting in cancer patients.

## Introduction

Sunitinib is an orally available tyrosine kinase inhibitor that potently inhibits vascular endothelial growth factor (VEGF) receptors (VEGFR1, VEGFR2 and VEGFR3), platelet derived growth factor (PDGF) receptors (PDGFRα and PDGFRβ) and several other receptor tyrosine kinases, including KIT receptor [1, 41, 46]. Extensive preclinical work shows that inhibitors of vascular endothelial growth factor signalling, such as sunitinib, can suppress tumour growth in mice by inhibiting tumour angiogenesis [2, 20, 25, 28, 32, 33, 48, 60]. Moreover, sunitinib has been shown to extend progression free survival and overall survival in patients with metastatic renal cell carcinoma (mRCC) and is now used as first line treatment for this disease [44, 45].

Despite these promising results, 20–30 % of mRCC patients show no response to sunitinib and even those that do respond initially will inevitably develop resistance and progress after several months of treatment [30, 45, 50]. This scenario is not unique, because similar findings are observed with VEGF-targeted agents in other indications. In trials of the VEGF neutralising antibody bevacizumab in metastatic breast, colorectal and lung cancer, only a subset of patients benefit from the combined use of bevacizumab with chemotherapy and the survival benefit afforded is measured only in terms of months or is not significant compared to chemotherapy alone [23, 35, 43, 51].

Importantly, preclinical studies are revealing mechanisms that allow tumours to exhibit intrinsic or acquired resistance to VEGF-targeted agents. These mechanisms include the stimulation of angiogenesis by alternative pro-angiogenic growth factors, the enhanced recruitment of pericytes or pro-angiogenic myeloid cells or the utilisation of alternative tumour vascularisation mechanisms such as vascular co-option [5, 7, 19, 38, 53]. In addition to these mechanisms of resistance, more recent work suggests that pharmacological inhibition of angiogenesis could also accelerate the growth of metastases. Treatment of several tumour models with VEGF receptor inhibitors, prior to resection of the primary tumour, lead to the increased incidence of distant metastasis in mice [13, 47, 52]. These data imply that the use of anti-angiogenic agents in the neoadjuvant setting could potentially promote the progression of metastases in patients. Further to this, administration of sunitinib after resection of the primary tumour increased the incidence of metastasis in mice [18]. In the same study, treatment of mice with sunitinib prior to, or after, intravenous injection of tumour cells also promoted the growth of metastases [18]. These data imply that anti-angiogenic agents could accelerate the growth of metastases both in the adjuvant setting and in patients with established metastatic disease. Although the analysis of large clinical studies currently provides no evidence for accelerated growth of metastasis in patients treated with clinically approved agents such as sunitinib or bevacizumab [21, 22, 39, 42], rapid tumour regrowth has been observed in some individuals after withdrawing anti-angiogenic therapy [6, 10].

Contradictory evidence therefore exists regarding the ability of VEGF-targeted agents to control the growth of metastasis. Here we examined the effect of sunitinib on the growth of metastases in mice when it was administered either prior to or following intravenous injection of tumour cells. We identify mouse models of metastasis that show contrasting responses to sunitinib and use these to examine the effects of this drug on tumour growth and outcome.

## Materials and methods

### Mice, reagents and cell lines

Female Balb/c mice were obtained from Charles River UK Ltd (Margate, Kent, UK). Sunitinib malate was obtained from LC laboratories (Woburn, MA, USA). Tissue culture reagents were obtained from Invitrogen Ltd (Paisley, Renfrewshire, UK), except for endothelial cell mitogen that was obtained from Serotech (Kidlington, Oxon., UK). Unless otherwise stated, all other reagents were obtained from Sigma (Poole, Dorset, UK). The 4T1 murine mammary carcinoma cell line that stably expresses the luciferin gene (4T1-luc) was obtained from Caliper Life Sciences (Runcorn, Cheshire, UK). The RENCA murine renal carcinoma cell line that stably expresses the luciferin gene (RENCA-luc) was prepared as previously described [60]. Tumour cell lines were cultured in RPMI medium supplemented with 10 % foetal calf serum (FCS) in 5 % CO_2_ at 37 °C. Human umbilical vein endothelial cells (HUVECs) from pooled donors (TCS Cell Works, Buckingham, Bucks., UK) were cultured in HUVEC-specific medium (M199 medium supplemented with 20 % FCS, 20 μg/ml endothelial cell mitogen, 10 μg/ml heparin and antibiotics) in 8 % CO_2_ at 37 °C. HUVECs were used for experiments at passage 4–8.

### Preparation of sunitinib for oral dosing

Vehicle for sunitinib consisted of 0.5 % w/v carboxymethylcellulose sodium, 1.8 % w/v NaCl, 0.4 % w/v Tween-80, 0.9 % w/v benzyl alcohol dissolved in reverse osmosis deionised water adjusted to pH 6.0. For oral dosing, sunitinib malate powder was added to vehicle and vortexed to create a suspension. This drug suspension was prepared at least 24 h before administration and stored at 4 °C in the dark. Fresh stocks of sunitinib suspension were prepared every week. Oral dosing in mice was performed by administration of 0.2 ml of vehicle or sunitinib suspension by oral gavage.

### In vivo tumour models and bioluminescence imaging

To examine the effect of sunitinib on the growth of lung metastases, female Balb/c mice at 8–10 weeks of age were injected intravenously with 4T1-luc or RENCA-luc tumour cells (2 × 10^5^ cells in 100 μl). Where pre-treatment was employed, mice were administered vehicle or sunitinib at the indicated dose by oral gavage every day for 7 days and cells were injected 24 h after the last dose was administered. Where treatment was administered after intravenous tumour cell injection, mice commenced treatment 24 h after injection with cells. Humane endpoints were used to measure the survival of tumour bearing mice in accordance with UK Home Office guidelines. Specifically, mice were culled when they developed any of the following clinical signs: loss of 20 % body mass, dypsnea, ataxia or seizure. For IVIS imaging, mice were injected intraperitoneally with 75 mg/kg D-luciferin (Caliper Life Sciences Hopkinton, MA), immediately anaesthetised with isofluorane and then imaged. Imaging was performed using a Lumina II™ IVIS (In Vivo Imaging System) instrument (Caliper Life Sciences) with quantification of bioluminescence performed using Living Image™ software (Caliper Life Sciences) according to manufacturers instructions.

### Measuring plasma concentrations of sunitinib

Female Balb/c mice at 8–10 weeks of age were treated with either a single dose of drug, or for seven days with drug (30, 60 or 120 mg/kg/day sunitinib) by oral gavage. At the appropriate time point, blood was collected by cardiac puncture under terminal anaesthesia. Blood samples were spun at 250 g for 10 min to isolate the plasma fraction, which was then stored in 100 μl aliquots at −20 °C until analysis. Calibration standards, ranging from 5 nM to 100 μM final sunitinib concentration, were prepared by spiking 100 μl samples of control mouse plasma with sunitinib. Calibration standards and test samples were precipitated with methanol, vortexed and then centrifuged at 21,000*g* for 30 min at 4 °C. The supernatant was then transferred to clean autosampler vials for subsequent analysis, which was performed on a quadrupole ion trap mass spectrometer with electrospray in positive ionisation mode (Thermo Scientific, Hemel Hempstead, Herts., UK).

### Tumour histology and immunohistochemistry

For ex vivo quantification of tumour burden, formalin fixed paraffin embedded sections of mouse lung were stained with haematoxylin and eosin (H&E) and then digitally scanned using an automated scanning microscope (Ariol system, Leica Microsystems Ltd, Milton Keynes, Bucks., UK). Tumour burden in the scanned images was measured using Adobe Photoshop image analysis software (Adobe, Uxbridge, Middx., UK). In brief, the marquee tool was used freehand to create regions of interest (ROIs) around areas of tumour in the section. The area of these ROIs was then calculated by the software and added together (t). The total area of the section (T) was measured using the same freehand ROI method. The percentage area of lung section that was occupied by tumour was then calculated according to this formula: (t/T) × 100. Lesion diameters were measured using the ruler tool in Adobe Photoshop.

Immunohistochemistry was performed in frozen tissue sections. At sacrifice, lungs were embedded in OCT on dry ice immediately following resection, followed by preparation of frozen sections (30 μM sections for analysis of tumour cell seeding and pericyte coverage in lung vasculature, 8 μM for all other studies). For staining, sections were brought to room temperature, fixed in 4 % w/v formalin for 10 min followed by incubation in blocking buffer (PBS supplemented with 1 % BSA and 10 μg/ml normal goat serum) for 1 h and then incubation with primary antibodies in blocking buffer overnight at 4 °C. Primary antibodies used were as follows: rat anti-CD31 (BD Biosciences, Oxford, Oxon., UK), rabbit anti-NG2 proteoglycan (Millipore, Billerica, MA), FITC-conjugated mouse anti-pimonidazole (HPI Inc., Burlington, MA), Alexa-488 conjugated rat anti-Gr1, phycoerythrin-conjugated rat anti-CD11b (Biolegend, San Diego, CA), biotin-conjugated rat anti-CD31 (BD Biosciences), rabbit anti-CAIX and rabbit anti-GLUT1 (Abcam, Cambridge, UK). After 3 × 5 min washes in PBS, sections were incubated with fluorescently conjugated secondary antibodies and/or fluorescently conjugated streptavidin and/or DAPI (Invitrogen Ltd) for 2 h at room temperature. After 3 × 5 min washes in PBS, sections were mounted with a glass coverslip in MOWIOL mounting solution supplemented with antifade (0.1 % w/v 1,4-diazabicyclo[2.2.2]octane).

Images were captured using a confocal laser scanning microscope (Leica) or the Ariol System automated scanning microscope (Leica). For studies of cell seeding in the lungs, cells were labeled with 10 μM carboxyfluorescein diacetate succinimidyl ester (Invitrogen) as per the manufacturers instructions just prior to intravenous injection. The number of fluorescent cells seeded per lung section was determined by counting using a fluorescence microscope. To determine pericyte coverage of vessels, confocal images of CD31 and NG2 staining were captured and the percentage of CD31 pixels that colocalised with NG2 pixels was calculated using a colocalisation plug-in for ImageJ. To determine microvessel density, the number of CD31 positive vessels present within the viable tissue in each tumour nodule was counted manually. For quantification of pimonidazole staining, mice were injected intraperitoneally with 60 mg/kg pimonidazole hydrochloride in 100 μl of saline at 1 h prior to sacrifice. To measure pimonidazole staining, Adobe Photoshop software was used to draw freehand ROIs around areas of positive staining, followed by automatic calculation of the area by the software. To determine the number of Gr1+/CD11b+ dual positive cells, Adobe Photoshop was used to create multi-channel images and cells labeling for both markers in each nodule were counted manually by an observer that was blinded. Areas were calculated in Adobe Photoshop using a freehand ROI method. These data were then used to calculate (a) the number of tumour cells seeded per mm^2^, (b) the microvessel density per mm^2^, (c) the % area of tumour that stained positive for pimonidazole, and (d) the number of Gr1/CD11b dual positive cells per mm^2^. On average, 20–40 tumour lesions were sampled from 6 to 8 mice to generate each data point.

### ^18^FDG-PET/CT imaging

Female Balb/c mice at 8–10 weeks of age were injected intravenously with 4T1-luc or RENCA-luc tumour cells (2 × 10^5^ cells in 100 μl). The following day, mice began treatment with vehicle or 60 mg/kg/day sunitinib on a continuous dosing schedule. Mice were imaged on the indicated days after tumour cell injection. On the day of imaging, mice were starved for at least 4 h prior to being anaesthetised and injected via the tail vein with 5–15 Mbq of ^18^FDG. This was then followed by a 90 min uptake period under continuous isoflurane anaesthesia before PET images were acquired. CT and PET scanning was performed using an Inveon microPET/CT scanner (Siemens, Munich, Germany). Isoflurane anaesthesia was continued for the duration of the scans (approximately 6 min for the attenuation CT and 20 min for the PET acquisition). Mice were maintained on a thermostatic heating pad during the entire anaesthesia period so as to maintain core body temperature. Inveon Acquisition Workplace software (Siemens) was used for image acquisition, whilst Cobra software (Exxim Computing Corporation, Pleasanton, CA) was used to reconstruct the CT images and Inveon Research Workplace software (Siemens) was used for reconstructing PET images and calculation of Standardized Uptake Values (SUV) according to manufacturers instructions. In order to calculate mean SUV in mouse lungs, Inveon Research Workplace software was used to place a 3-dimensional region of interest (ROI) in the area corresponding to the left lung or the right lung of each mouse, taking care to ensure that the ROIs were not placed in the region of the heart. The radioactivity concentration measured within the ROIs was then normalized to the amount of injected activity and the weight of the mice when calculating the SUV.

### Post-mortem measurement of ^18^FDG uptake

Female Balb/c mice at 8–10 weeks of age were injected intravenously with 4T1-luc or RENCA-luc tumour cells (2 × 10^5^ cells in 100 ml). The following day, mice began treatment with vehicle or 60 mg/kg/day sunitinib on a continuous dosing schedule. After 14 days (4T1 model) or 19 days (RENCA model), mice were starved for at least 4 h prior to being anaesthetized and injected via the tail vein with 0.5–2 Mbq of ^18^FDG. This was then followed by a 90 min uptake period under continuous isofluorane anaesthesia, and thermostatic heating, before mice were culled. Lungs, blood and muscle tissue were collected, weighed and then counted immediately in a gamma counter (Perkin Elmer, Cambridge, Camb., UK). Counts were corrected to the tissue mass in order to calculate the percentage of injected dose present per gram of tissue.

### In vitro cell proliferation assays

Human umbilical vein endothelial cells (HUVECs) were plated on 96 well plates at a density of 1,000 cells/well. The next day the medium was changed for M199 plus 10 % fetal calf serum, supplemented with 100 ng/ml VEGF (R&D Systems, Abingdon, Oxon., UK) and with sunitinib at the indicated concentration or vehicle (0.1 % DMSO). 4T1 cells or RENCA cells were plated on 96 well plates at a density of 1,000 cells/well. The next day the medium was changed for M199 plus 10 % fetal calf serum, supplemented with sunitinib at the indicated concentration or vehicle (0.1 % DMSO). After 72 h, cell viability was quantified using the Cell-TitreGlo cell viability reagent (Promega, Southampton, Hants., UK) according to the manufacturers instructions. The plates were read in a luminescence plate reader (PerkinElmer).

### Ethical approval for animal experimentation

Ethical approval for animal experimentation was granted by: the Institute of Cancer Research Animal Ethics Committee and the Queen Mary University of London Animal Ethics Committee. All procedures were performed in accordance with UK Home Office regulations.

### Statistical analysis

Statistical analysis of data was performed using Student’s *t* test, except for the analysis of the Kaplan–Maier survival data, which was performed using the Log-rank test. *P* values below 0.05 were considered to be significant.

## Results

### The ability of sunitinib to enhance the seeding of metastasis is cell line dependent

**Fig. 1:**
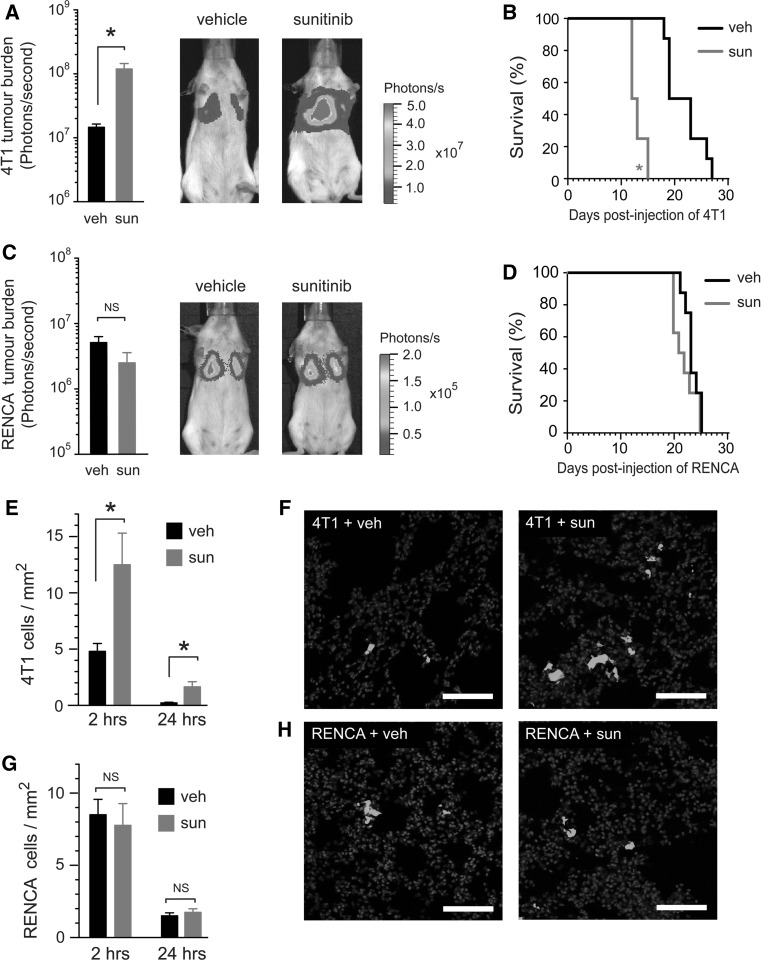
The ability of sunitinib to enhance the seeding of metastasis is cell line dependent. **a**–**d** Quantification of tumour burden and survival. Balb/c mice were pre-treated with vehicle (veh) or 120 mg/kg/day sunitinib (sun) every day for 7 days, followed by intravenous injection of luciferase-tagged 4T1 tumour cells (4T1-luc) or luciferase-tagged RENCA tumour cells (RENCA-luc). In **a** and **c**, *bar graphs* show bioluminescence signal ± SEM at 12 days post tumour cell injection in 4T1-luc (**a**) or RENCA-luc (**c**) tumour bearing mice. Representative bioluminescence images of mice at 12 days post tumour cell injection are also shown. *P = 0.0007, n = 8 mice per treatment group. In **b** and **d**, Kaplan–Meier analysis of overall survival in 4T1-luc (**b**) or RENCA-luc (**d**) tumour bearing mice is shown. *P = 0.0009, n = 8 mice per treatment group. **e**–**h** Quantification of tumour cell seeding in the lungs. Balb/c mice were pre-treated with vehicle (veh) or 120 mg/kg/day sunitinib (sun) every day for 7 days, followed by intravenous injection of fluorescently labeled 4T1-luc or RENCA-luc cells. In **e** and **g**, *bar graphs* show number of tumour cells counted per mm^2^ of lung tissue section ± SEM at 2 and 24 h after injection of 4T1-luc (**e**) or RENCA-luc (**g**). *P = 0.02, n = 8 mice per treatment group. Representative images of lung sections from 4T1-luc (**f**) or RENCA-luc (**h**) tumour bearing mice are also shown, tumour cells (*green*) and DAPI (*blue*). *Scale bar* = 100 μM. NS = no significant difference. (Color figure online)

Daily administration of sunitinib at a dose of 120 mg/kg/day for 7 days prior to intravenous injection of tumour cells has been reported to promote the growth of metastases in mice and leads to a shortening of overall survival. These data suggest that “pre-conditioning” of mice with sunitinib can promote the formation of metastases by circulating tumour cells [18]. We began our study by performing similar experiments using two syngeneic murine tumour cell lines. Balb/c mice were pre-treated for 7 days with vehicle or 120 mg/kg/day sunitinib, followed by intravenous injection of luciferase-tagged 4T1 tumour cells (4T1-luc) or luciferase-tagged RENCA tumour cells (RENCA-luc). In mice inoculated with 4T1-luc cells, sunitinib pre-treatment resulted in significantly enhanced lung tumour burden (*P* = 0.0007) and significantly shortened overall survival (*P* = 0.0009) compared to vehicle treated controls (Fig. 1a, b). However, lung tumour burden and overall survival in mice injected with RENCA-luc cells was equivalent in both the vehicle and sunitinib treated groups (Fig. 1c, d).

We next addressed whether sunitinib increases lung tumour burden by promoting the seeding of tumour cells in the lungs of mice. Balb/c mice were pre-treated for 7 days with vehicle or 120 mg/kg/day sunitinib, followed by intravenous injection of 4T1 or RENCA tumour cells that had been fluorescently labelled just prior to injection. Lungs were harvested at 2 or 24 h post-injection to assess the seeding of metastasis in the lungs by fluorescence microscopy. At 2 h post-injection, significantly more 4T1 tumour cells were counted in the lungs of sunitinib treated mice compared to vehicle controls (Fig. 1e, f). By 24 h, the number of cells in the lungs in both treatment groups had reduced significantly, which is a well-described feature of such lung metastasis models and is due to the apoptosis of tumour cells [62]. However, even after this attrition, enhanced numbers of 4T1 tumour cells survived in the lungs of sunitinib pre-treated mice, compared to vehicle controls at the 24 h time point (Fig. 1e). In contrast, assessment of RENCA cells in the lungs of mice revealed no significant difference in cell numbers between vehicle and sunitinib pre-treated mice at either time point (Fig. 1g, h). These data indicate that sunitinib can increase lung tumour burden by promoting the seeding of metastasis, but that this effect is cell line dependent.

### Reduced pericyte coverage in the lung microvasculature is associated with enhanced seeding of metastasis

**Fig. 2:**
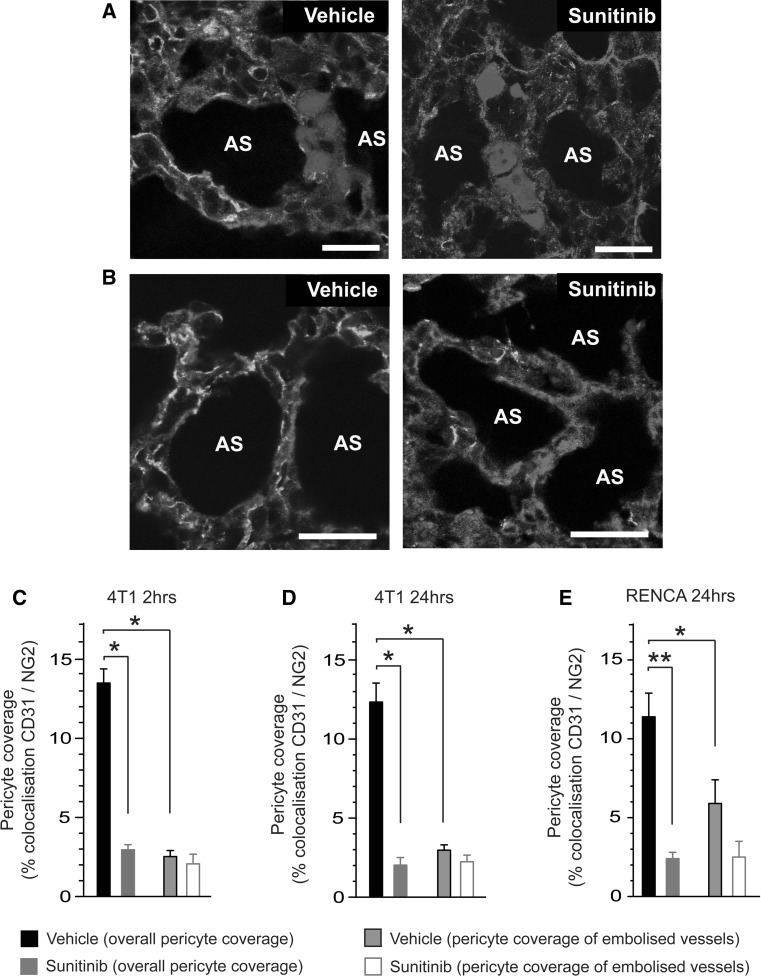
Reduced pericyte coverage in the lung microvasculature is associated with enhanced seeding of metastasis. Balb/c mice were pre-treated with vehicle (veh) or 120 mg/kg/day sunitinib (sun) every day for 7 days, followed by intravenous injection of fluorescently tagged 4T1-luc or RENCA-luc tumour cells. Lungs were harvested at 2 or 24 h post-injection of tumour cells and sections were stained for the vessel marker CD31 and the pericyte marker NG2. **a** Representative images of 4T1 tumour cell emboli trapped within the lung vasculature at 24 h post-injection of tumour cells in mice pre-treated with vehicle or sunitinib as indicated, tumour cells (*blue*), CD31 (*red*) and NG2 (*green*). **b** Representative images of pericyte coverage within the lung vasculature at 24 h post-injection of 4T1 tumour cells in mice pre-treated with vehicle or sunitinib as indicated, CD31 (*red*), NG2 (*green*) and DAPI (*blue*). **c**, **d** Quantification of overall pericyte coverage in the lung vasculature and pericyte coverage only in vessels containing 4T1 tumour cell emboli. Data are shown at 2 h (**c**) and 24 h (**d**) post-injection of tumour cells in mice pre-treated with vehicle or sunitinib. *Bar graph* shows percentage colocalisation between CD31 and NG2 ± SEM. * *P* = 0.0001, n = 8 mice per treatment group. **e** Quantification of overall pericyte coverage in the lung vasculature and pericyte coverage only in vessels containing RENCA tumour cell emboli. Data are shown at 24 h post-injection of tumour cells in mice pre-treated with vehicle or sunitinib. *Bar graph* shows percentage colocalisation between CD31 and NG2 ± SEM. * *P* = 0.03, ** *P* = 0.0001. n = 8 mice per treatment group. *Scale bar* = 25 μM, AS = alveolar space. (Color figure online)

Lung tissue sections obtained from the previous experiment were stained for the vessel marker CD31 and the pericyte marker NG2. Careful examination of these tissue sections revealed that, at both 2 and 24 h post-injection of tumour cells, 4T1 cells remained trapped as tumour emboli within the lung microvasculature in both vehicle and sunitinib pre-treated mice (for example see Fig. 2a). These data are consistent with previous work showing that 4T1 tumour cells form intravascular colonies in the lungs of mice and that extravasation occurs only when micrometastatic foci outgrow the vessels they are in [63]. We noted also that pericyte coverage of the microvasculature was clearly reduced in the lungs of sunitinib pre-treated mice compared to vehicle controls (for example see Fig. 2a, b). We then examined the relationship between pericyte coverage and the seeding of 4T1 tumour cells. First, quantification of overall pericyte coverage in the lung microvasculature revealed that pericyte coverage overall was significantly reduced in the lungs of sunitinib treated mice compared to vehicle controls at both time points (Fig. 2c, d). Second, we quantified pericyte coverage specifically in vessels that contained tumour cell emboli. In vehicle pre-treated mice, vessels containing 4T1 tumour cell emboli had significantly reduced pericyte coverage compared to the mean overall pericyte coverage of the lung microvasculature (Fig. 2c, d). Indeed, the pericyte coverage in these embolised vessels was equivalent to the mean overall pericyte coverage observed in the lungs of sunitinib pre-treated mice (Fig. 2c, d). In parallel, examination of lungs from mice injected with RENCA tumour cells revealed that RENCA tumour cell emboli were also preferentially localised to vessels with lower pericyte coverage (Fig. 2e). However, the difference between overall pericyte coverage and pericyte coverage of embolised vessels was less marked for RENCA cells than it was for 4T1 cells (compare Fig. 2d with Fig. 2e). These data show that sunitinib can deplete pericyte coverage in the lung microvasculature and that 4T1 emboli, but to a lesser extent RENCA emboli, preferentially seed in the lungs at sites of reduced pericyte coverage.

### Enhanced seeding of metastasis is sunitinib dose-dependent

**Fig. 3:**
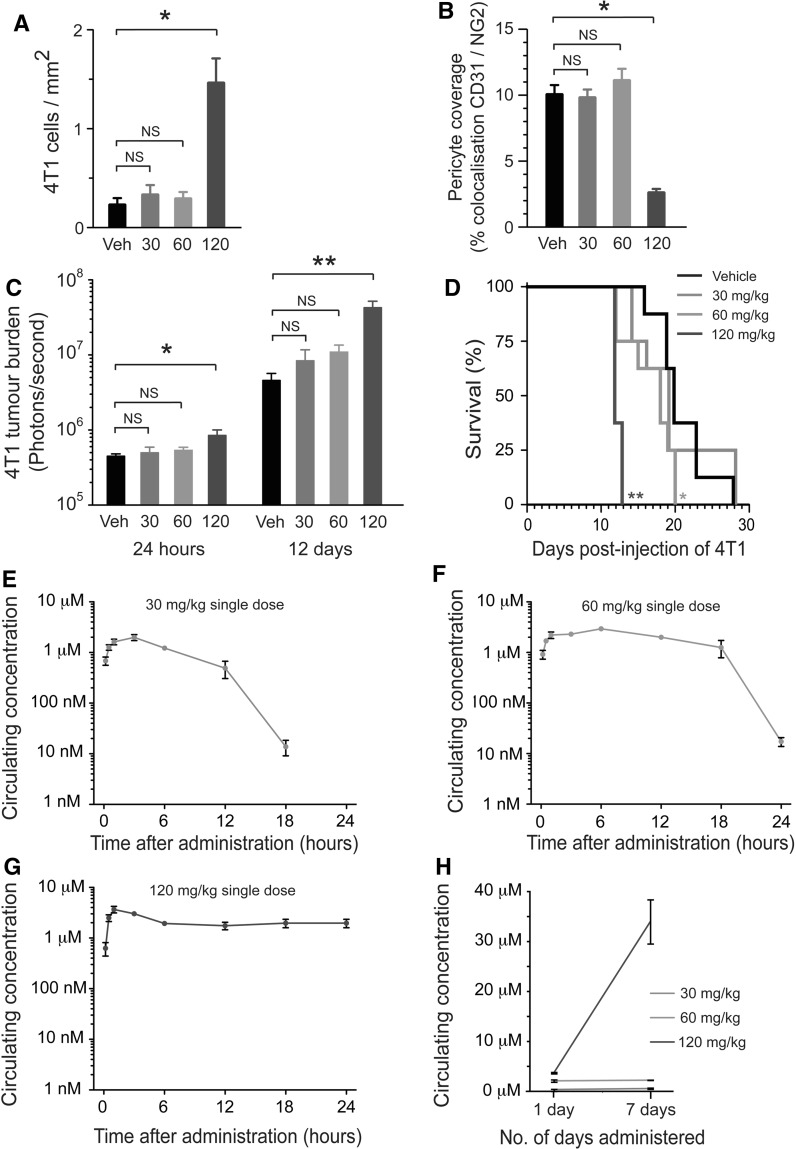
Enhanced seeding of metastasis is sunitinib dose-dependent. **a**–**d** Dose-dependent effects of sunitinib. Balb/c mice were pre-treated with 30, 60 or 120 mg/kg/day sunitinib or vehicle for 7 days, followed by intravenous injection of 4T1-luc tumour cells. **a** Quantification of tumour cells seeded in the lung at 24 h post tumour cell injection in mice pre-treated with the indicated dose of sunitinib. *Bar graph* shows number of cells counted per mm^2^ of lung tissue section ± SEM. *P = 0.01, n = 8 mice per treatment group. **b** Quantification of pericyte coverage in the normal lung vasculature at 24 h post-injection of tumour cells in mice pre-treated with the indicated dose of sunitinib. *Bar graph* shows percentage colocalisation between CD31 and NG2 ± SEM. *P = 0.0001, n = 8 mice per treatment group. **c** Bioluminescence signal at 1 day or 12 days post tumour cell injection in mice pre-treated with the indicated dose of sunitinib ± SEM, *P = 0.01, **P = 0.002, n = 8 mice per treatment group. **d** Kaplan–Meier analysis of overall survival is shown in mice pre-treated with the indicated dose of sunitinib. *P = 0.03, **P = 0.0001, n = 8 mice per group. **e**–**h** Pharmacokinetics of sunitinib. Balb/c mice were administered a single oral dose of 30 mg/kg (**e**), 60 mg/kg (**f**) or 120 mg/kg (**g**) sunitinib. At the indicated times after dosing, blood samples were collected and the plasma concentration of sunitinib was determined. n = 4 mice per time point. **h** Balb/c mice were administered a single dose of sunitinib at 30, 60 or 120 mg/kg or were administered sunitinib at 30, 60 or 120 mg/kg every day for 7 days. At 12 h after administration of a single sunitinib dose, or at 12 h after administration of the seventh dose of sunitinib, blood samples were collected and the plasma concentration of sunitinib was determined. n = 4 mice per time point, NS = no significant difference

The dose of 120 mg/kg/day sunitinib which increased lung tumour burden here, and in a previous study [18], is a relatively high dose considering that doses in the range of 20–60 mg/kg/day sunitinib are typically sufficient to suppress tumour growth and extend survival in tumour bearing mice [17, 33, 41]. We therefore proceeded to examine the outcome when mice were pre-treated with lower sunitinib doses. Balb/c mice were pre-treated for 7 days with 30, 60 or 120 mg/kg/day sunitinib or vehicle alone, followed by intravenous injection of 4T1-luc cells. Histological examination of the lungs at 24 h post-inoculation demonstrated significantly enhanced seeding of 4T1 cells in mice pretreated with 120 mg/kg/day sunitinib compared to vehicle controls, but not in mice pretreated with 30 or 60 mg/kg/day (Fig. 3a). In concordance with this finding, we observed significantly reduced pericyte coverage in the lungs of mice pretreated with 120 mg/kg/day sunitinib compared to vehicle controls, but not in mice pretreated with 30 or 60 mg/kg/day sunitinib (Fig. 3b). Bioluminescence imaging revealed that although lung tumour burden in mice pretreated with 120 mg/kg/day sunitinib was significantly increased compared to vehicle-treated controls at 24 h (*P* = 0.01) and 12 days (*P* = 0.002) after tumour cell injection, tumour burden in the lungs of mice pretreated with 30 or 60 mg/kg/day sunitinib was not statistically different to vehicle treated mice at either time point (Fig. 3c). In terms of overall survival, in mice pretreated with 120 mg/kg/day sunitinib we again observed shortened survival compared to vehicle (median survival 12 days vs. 20 days, *P* = 0.0001, Fig. 3d). Survival was also shortened, albeit to a lesser extent, by pre-treatment with 60 mg/kg/day sunitinib compared to vehicle (median survival 18 days vs. 20 days, *P* = 0.03, Fig. 3d), but pre-treatment with 30 mg/kg/day sunitinib did not significantly shorten survival compared to vehicle (median survival 19 days vs. 20 days; *P* = 0.7, Fig. 3d).

We then examined the pharmacokinetics of sunitinib when administered at different doses (Fig. 3e–g). All three doses (30, 60 and 120 mg/kg) achieved a similar C_max_ of ≥2 μM within a few hours of administration to mice. However, the clearance of sunitinib was markedly different for the three doses. Whilst clearance of drug began at 8 h post-administration in mice treated with 30 mg/kg sunitinib and dipped below the limit of detection by 24 h (Fig. 3e), clearance in mice treated with 60 mg/kg sunitinib was less efficient (Fig. 3f). Importantly, in mice treated with 120 mg/kg sunitinib, no significant clearance was evident even after 24 h (Fig. 3g). We then addressed whether the inefficient clearance of sunitinib observed at higher doses could result in elevated circulating levels of sunitinib after prolonged dosing. After 7 days treatment with 120 mg/kg/day sunitinib, circulating concentrations of drug were elevated to ~30 μM, whereas no such increase was observed in mice treated for 7 days with 30 or 60 mg/kg/day sunitinib (Fig. 3h). These data suggest that the enhanced seeding of metastasis induced by sunitinib occurs only when mice are exposed to sustained micromolar circulating concentrations of the drug.

### Contrasting outcomes in mice administered sunitinib after tumour cell seeding in the lungs

**Fig. 4:**
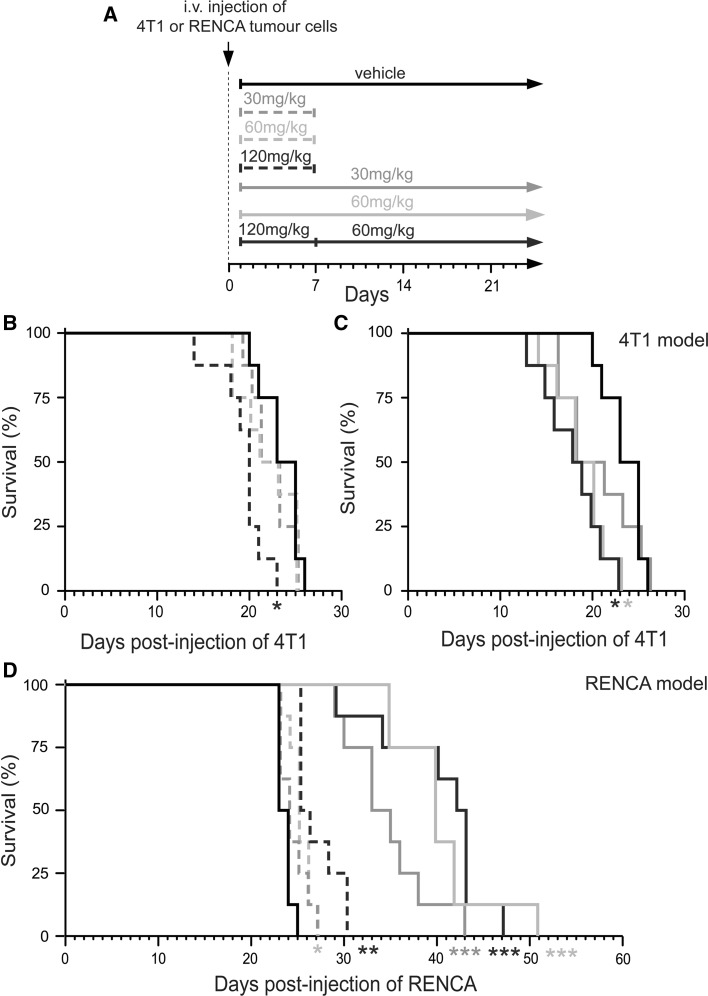
Survival of mice treated with sunitinib after intravenous injection of tumour cells. **a** Schematic diagram of dosing schedules tested. Balb/c mice were injected intravenously with 4T1-luc or RENCA-luc tumour cells and then treated with vehicle, 30, 60 or 120 mg/kg/day sunitinib daily for only 7 days (short term therapy) or daily until they became moribund (continuous dosing schedule). **b**, **c** Kaplan–Meier analysis of survival for 4T1-luc tumour bearing mice treated with vehicle or sunitinib under the dosing schedules shown in **a**. The *two graphs* show results from the same experiment, but survival of mice treated with sunitinib for 7 days (**b**) or treated with sunitinib continuously (**c**) have been presented as separate graphs for clarity. *P = 0.003, n = 8 mice per treatment group. **d** Kaplan–Meier analysis of survival for RENCA-luc tumour bearing mice treated with vehicle or sunitinib under the dosing schedules shown in **a**. *P = 0.01, **P = 0.0004, ***P = 0.0001, n = 8 mice per treatment group

We then proceeded to examine tumour growth and outcome when sunitinib was administered after intravenous inoculation of tumour cells. Balb/c mice were injected intravenously with 4T1-luc or RENCA-luc tumour cells. After 24 h, these mice then commenced treatment with vehicle or sunitinib. Mice were administered sunitinib at different doses and under various schedules (see Fig. 4a). We tested short term therapy with 30, 60 or 120 mg/kg/day sunitinib, where drug was administered daily for 1 week only. We also tested continuous therapy where mice were administered daily with 30 or 60 mg/kg/day sunitinib until they became moribund. In addition we tested treatment for 7 days with 120 mg/kg/day sunitinib, followed by switching to continuous therapy with 60 mg/kg/day sunitinib which was administered until mice became moribund (120–60 schedule). Importantly, none of these dosing regimes were capable of extending overall survival in 4T1 tumour bearing mice (Fig. 4b, c). Indeed, significantly shortened overall survival compared to vehicle controls was observed in mice receiving short term therapy with 120 mg/kg/day sunitinib (median survival 20 days vs. 23 days; *P* = 0.003), continuous therapy with 60 mg/kg/day sunitinib or the 120–60 schedule (median survival 18 days vs. 23 days; *P* = 0.003). In contrast, sunitinib was able to significantly extend survival compared to vehicle controls in RENCA tumour bearing mice (Fig. 4d). Continuous dosing was particularly effective in the RENCA model, with median survival extended to 33 days or beyond, compared to a median survival of 23 days in vehicle-treated mice (Fig. 4d). These data show that whilst sunitinib can shorten overall survival in 4T1 tumour bearing mice, the same drug can extend survival in RENCA tumour bearing mice.

**Fig. 5:**
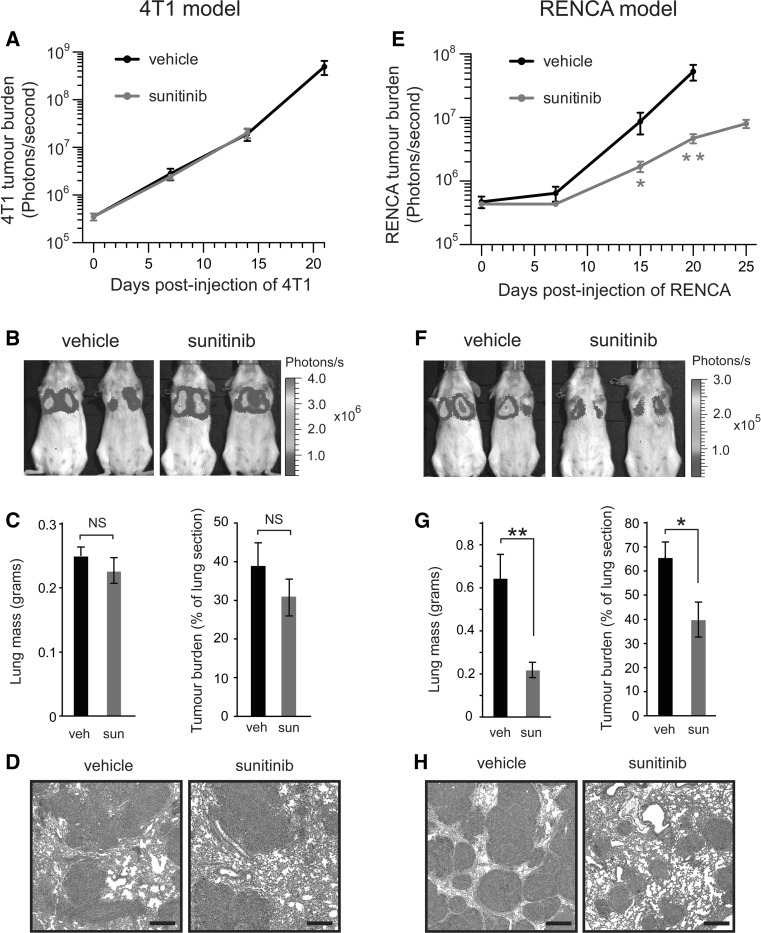
Lung tumour burden in mice treated with sunitinib after intravenous injection of tumour cells. Balb/c mice were injected intravenously with 4T1-luc or RENCA-luc tumour cells and then treated with daily vehicle (veh) or 60 mg/kg/day sunitinib (sun) on a continuous dosing schedule. **a**, **e** Lung tumour burden was quantified in 4T1-luc (**a**) or RENCA-luc (**e**) tumour bearing mice. *Graphs* show bioluminescence signal at the indicated time points after injection of tumour cells ± SEM. Note that the difference in duration over which bioluminescence is monitored for each experimental group is due to the difference in the overall survival observed in each group. *P = 0.02, **P = 0.004, n = 8 mice per treatment group. **b**, **f** Representative bioluminescence images of 4T1-luc (**b**) or RENCA-luc (**f**) tumour bearing mice at 14 days (4T1-luc) or 15 days (RENCA-luc) after tumour cell injection. **c**, **g** Tumour burden was quantified in 4T1-luc (**c**) or RENCA-luc (**g**) tumour bearing mice at 14 days (4T1-luc) or 18 days (RENCA-luc) after tumour cell injection by weighing lungs or by quantitative histology. *Graphs* show lung mass ± SEM or percentage area of lung section occupied by tumour ± SEM. *P = 0.01, **P = 0.0001, n = 8 mice per treatment group. **d**, **h** Representative fields of H&E stained sections of lung from 4T1-luc (**d**) or RENCA-luc (**h**) tumour bearing mice at 14 days (4T1-luc) or 18 days (RENCA-luc) after tumour cell injection. *Scale bar* = 400 μM, NS = no significant difference

To examine the effect of sunitinib treatment on tumour growth in the two metastasis models, lung tumour burden was examined using three methods: in vivo bioluminescence imaging, weighing freshly resected lungs and quantitative histology of lung sections. We chose to use continuous therapy with 60 mg/kg/day sunitinib, because this regimen gave rise to the most contrasting difference in responses i.e. significantly shortened overall survival in the 4T1 model (Fig. 4c) and significantly increased overall survival in the RENCA model (Fig. 4d). Surprisingly, lung tumour burden in sunitinib treated mice was not significantly different from vehicle treated controls in the 4T1 model (Fig. 5a–d). By contrast, in the RENCA model, sunitinib treatment resulted in significantly decreased lung tumour burden compared to vehicle controls (Fig. 5e–h). Therefore, while sunitinib fails to inhibit the growth of 4T1 tumours after they have seeded in the lungs of mice, sunitinib can significantly suppress the growth of RENCA tumours.

**Fig. 6:**
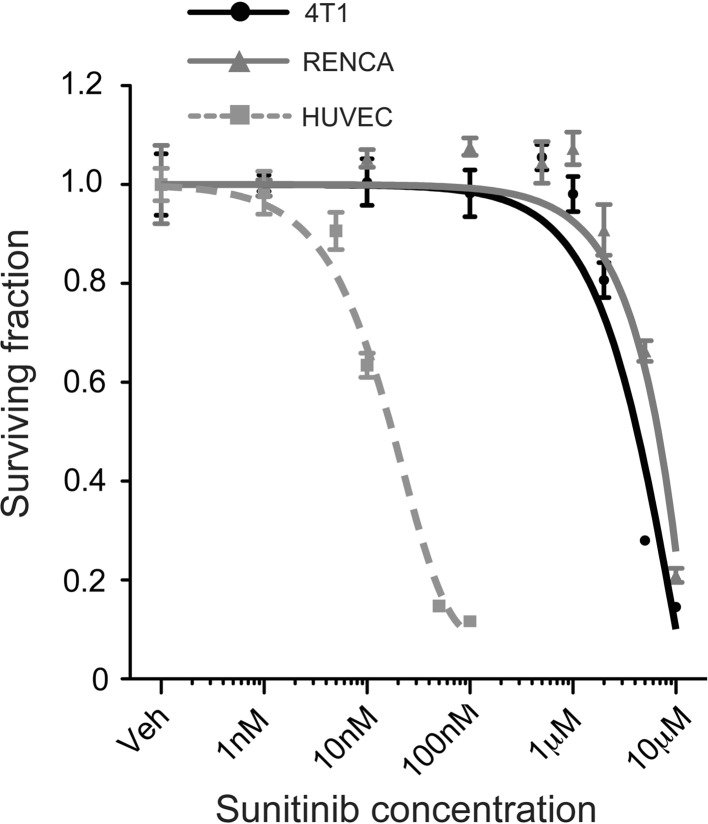
Effect of sunitinib on tumour cell viability. **a** The ability of 4T1 tumour cells, RENCA tumour cells or primary endothelial cells (HUVECs), to proliferate in the presence of sunitinib was measured in vitro. *Graphs* show the surviving fraction of cells after 72 h ± SEM

Since sunitinib is a relatively broad spectrum tyrosine kinase inhibitor, it could potentially target both the tumour vasculature and tumour cell proliferation directly [29]. Therefore, we examined the sensitivity of 4T1 and RENCA cells to sunitinib in vitro (Fig. 6). The IC50 for both cell lines was relatively high and very similar, with 4T1 cells (IC50 = 3 μM) being marginally more sensitive to sunitinib than RENCA cells (IC50 = 5 μM). As a positive control for sunitinib activity, we show that VEGF-induced proliferation of endothelial cells is 2 orders of magnitude more sensitive to sunitinib than the tumour cell lines (IC50 = 10 nM, Fig. 6). These data suggest that the superior efficacy of sunitinib in the RENCA model compared to the 4T1 model is not due to direct inhibition of RENCA cell proliferation by sunitinib.

### Analysis of microvessel density and hypoxia in lung metastases treated with sunitinib

**Fig. 7:**
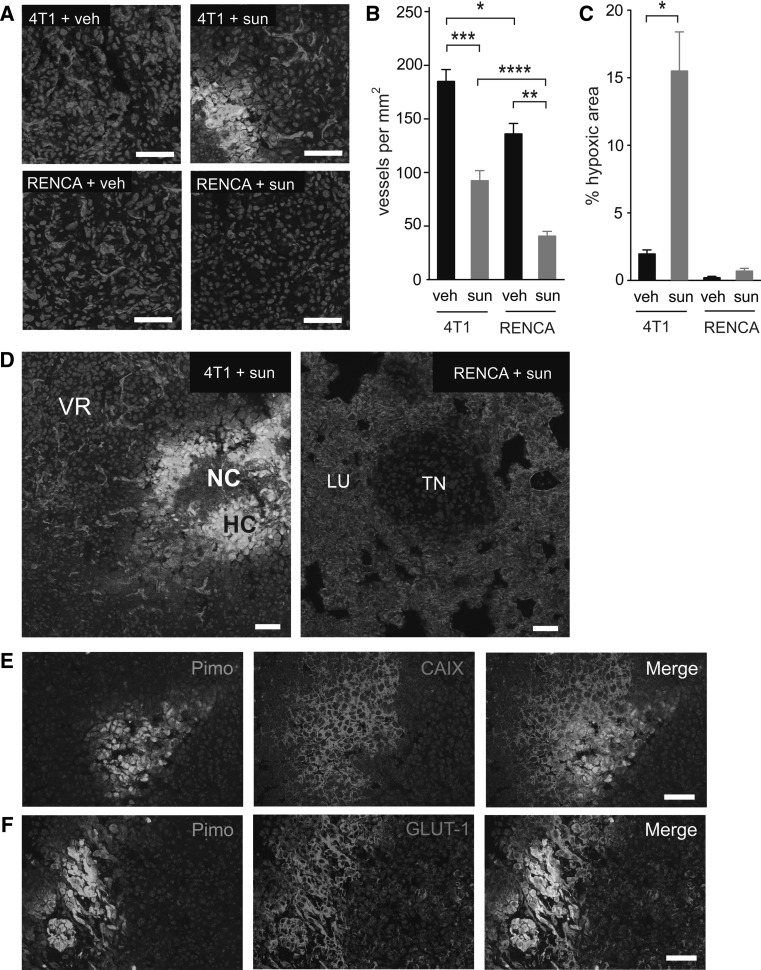
Quantification of tumour vascularisation and tumour hypoxia. Balb/c mice were injected intravenously with 4T1 or RENCA cells and then treated with vehicle (veh) or 60 mg/kg/day sunitinib (sun) for 14 days, at which time point mice were culled and lungs were collected for histological analysis. **a** Representative fields of CD31 (*red*), pimonidazole (*green*) and DAPI (*blue*) staining in 4T1 and RENCA tumour nodules from vehicle or sunitinib treated mice as indicated. **b** Quantification of microvessel density in tumour nodules. *Graph* shows number of vessels per mm^2^ ± SEM. *P = 0.015, **P = 0.008, ***P = 0.0003, ****P = 0.0004, n = 8 mice per treatment group. **c** Quantification of pimonidazole staining in tumour nodules. *Graph* shows percentage area of the tumour nodules that stained positive for pimonidazole ± SEM. *P = 0.001, n = 8 mice per treatment group. **d** *Left panel* low power view of a 4T1 lung tumour nodule from a sunitinib treated mouse. *Right panel* low power view of a RENCA lung tumour nodule from a sunitinib treated mouse. Staining is for CD31 (*red*), pimonidazole (*green*) and DAPI (*blue*). VR, vascular rim; HC, hypoxic core; NC, necrotic core; TN, tumour nodule; LU, normal lung. **e**, **f** Co-staining for pimonidazole and CAIX (**e**) or GLUT-1 (**f**) in 4T1 tumours. NS = no significant difference, *Scale bar* = 50 μM. (Color figure online)

We then addressed the effect of sunitinib on tumour angiogenesis and tumour hypoxia. Mice were injected intravenously with 4T1-luc or RENCA-luc cells and then commenced treatment with vehicle or 60 mg/kg/day sunitinib. After 2 weeks of treatment, lungs were harvested for histological examination. Staining for CD31 was used to assess tumour microvessel density (MVD) and the hypoxia probe pimonidazole hydrochloride was used to assess tumour hypoxia (Fig. 7a–c). Intratumoural vessels were present in both the 4T1 and RENCA lung tumour nodules, although MVD in the 4T1 tumour nodules was ~25 % higher than in RENCA tumours (*P* = 0.015). Importantly, sunitinib treatment led to a significant reduction in MVD in both the 4T1 and RENCA lung tumour nodules compared to the corresponding vehicle control tumours (*P* = 0.0003 for 4T1, *P* = 0.0004 for RENCA, Fig. 7b). However, sunitinib had a more profound effect on angiogenesis in the RENCA model than the 4T1 model. Whilst sunitinib treated RENCA tumours were reduced in MVD by ~70 % compared to vehicle controls, a reduction of only ~50 % was observed in the 4T1 model. In concordance with this, a significant difference between the MVD in sunitinib treated RENCA tumours and sunitinib treated 4T1 tumours was observed (*P* = 0.008, Fig. 7b).

Quantification of pimonidazole staining demonstrated that hypoxia was significantly increased in 4T1 tumours in sunitinib treated mice compared to vehicle treated controls (*P* = 0.001, Fig. 7a, c). Moreover, the 4T1 tumours that grew in sunitinib treated-mice typically consisted of two distinct zones. A well-vascularised zone of tumour cells was present around the rim of the tumours and a poorly-vascularised zone composed of pimonidazole-positive cells and necrotic tumour cells was present at the centre of the tumours (Fig. 7d, left hand image). These pimonidazole-positive regions also stained strongly positive for CAIX and GLUT-1 (Fig. 7e, f), markers that are synonymous with increased tumour hypoxia and increased tumour glucose metabolism [3, 8]. This observation is consistent with previous reports showing that impairment of vascular function caused by inhibition of VEGF signalling can lead to increased tumour hypoxia and the induction of hypoxia-associated genes [14, 27]. In contrast, hypoxia was not exacerbated in RENCA tumours in sunitinib-treated mice (Fig. 7a, c). Measurement of lesion diameters (see Table 1) revealed that RENCA lesions in the sunitinib treatment group were on average half the size of 4T1 lesions in sunitinib treated mice (464 ± 22 μm vs. 1,142 ± 48 μm). Since the diffusion limit of oxygen in vivo is estimated to be within the range of 200–250 μm [59] the RENCA lesions in sunitinib treated mice are likely to receive adequate oxygenation from the surrounding lung parenchyma, which may explain the lack of increased hypoxia in these RENCA tumours.

**Table 1:** Lesion diameter in each experimental group

	Treatment received	
Cohort	Vehicle (μm)	60 mg/kg/day sunitinib (μm)
4T1	1,065 ± 51	1,142 ± 48
RENCA	1,000 ± 40	464 ± 22

Mice were inoculated intravenously with 4T1-luc or RENCA-luc tumour cells and then treated continuously with daily vehicle or daily 60 mg/kg/day sunitinib. After 2 weeks lungs were harvested, H&E stained sections prepared and lesion diameters were measured microscopically. 100 lesions from each experimental group were measured. Lesion diameter in μm ± SEM is shown. n = 8 mice per experimental group

### Analysis of pericyte coverage and myeloid cell recruitment in lung metastases treated with sunitinib

**Fig. 8:**
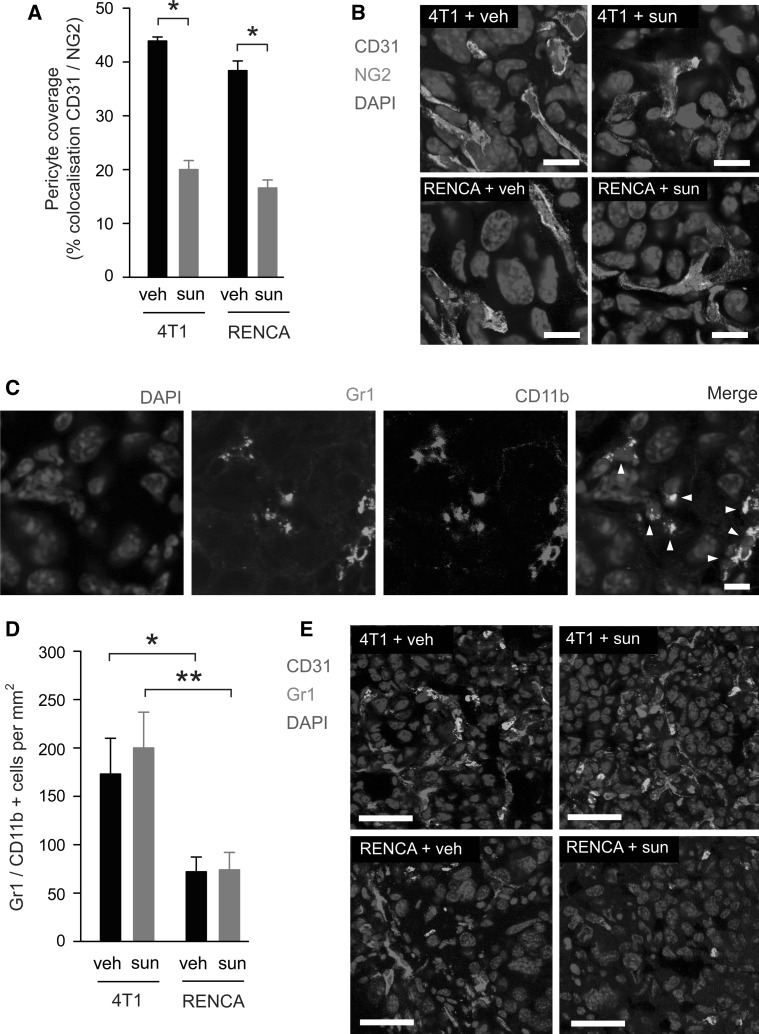
Quantification of pericyte coverage and myeloid cell recruitment in tumours. Balb/c mice were injected intravenously with 4T1-luc or RENCA-luc cells and then treated with vehicle (veh) or 60 mg/kg/day sunitinib (sun) for 14 days, at which time point mice were culled and lungs were collected for histological analysis. **a**, **b** Quantification of pericyte coverage in 4T1 and RENCA tumours treated with vehicle or sunitinib. *Bar graph* in **a** shows percentage colocalisation between CD31 and NG2 ± SEM. * *P* = 0.0001, n = 8 mice per treatment group. In **b**, representative high power fields from each experimental group are shown with CD31 (*red*), NG2 (*green*) and DAPI (*blue*). **c** Representative high power field containing Gr1 and CD11b dual positive cells (*arrowheads*). Staining for Gr1 (*green*), CD11b+ (*red*) and DAPI (*blue*). **d**, **e** Quantification of Gr1 and CD11b dual positive cells in 4T1 and RENCA tumour nodules from vehicle and sunitinib treated mice. *Graph* in **d** shows number of Gr1/CD11b+ cells per mm^2^ ± SEM. *P = 0.02, **P = 0.007, n = 8 mice per treatment group. In **e**, representative low power fields from each experimental group are shown with CD31 (*red*), Gr1 (*green*) and DAPI (*blue*). CD11b staining is omitted in **e**, but all of the cells shown were confirmed to be dual positive for both Gr1 and CD11b. *Scale bar* = 20 μM in **b** and **c** and 50 μM in **e**. (Color figure online)

Since sunitinib is a potent inhibitor of PDGF receptor signalling [1], and signalling by PDGF is an important mediator of pericyte recruitment during tumour angiogenesis [4], we examined pericyte coverage in each experimental group. Similar levels of pericyte coverage were observed in 4T1 and RENCA tumours from vehicle treated mice (Fig. 8a, b). However, sunitinib treatment resulted in a significant reduction in pericyte coverage of ~50 % in both tumour models (Fig. 8a, b). Therefore, we did not observe contrasting effects of sunitinib on pericyte coverage in 4T1 and RENCA tumours.

The ability of tumours to recruit and retain pro-angiogenic Gr1+CD11b+ myeloid cells has been implicated in both the induction of tumour angiogenesis [65] and in mediating resistance to anti-angiogenic therapies [54]. By co-staining sections of lung tissue with both Gr1- and CD11b-specific antibodies, we were able to identify myeloid cells within the tumour nodules that labeled positive for both Gr1 and CD11b (Fig. 8c). We then addressed whether the recruitment of these Gr1+CD11b+ myeloid cells differed between experimental groups. Moreover, by co-staining for CD31, we examined the proximity of these cells to intratumoural blood vessels. Significantly more Gr1+CD11b+ myeloid cells were present in 4T1 tumour nodules compared to RENCA tumours in both the presence and absence of sunitinib (Fig. 8d, e). These differences were not a function of tumour size, since we sampled 4T1 and RENCA tumours of a similar size range in order to determine these data. Interestingly, whilst Gr1+CD11b+ myeloid cells were typically colocalised with CD31 positive blood vessels in 4T1 tumours, this colocalisation was absent in RENCA tumours (Fig. 8e). These data suggest that 4T1 and RENCA lung metastases differ in their ability to recruit and retain pro-angiogenic myeloid cells, and that their capacity to recruit these cells is unaffected by sunitinib treatment.

### Measurement of glucose uptake in the lungs of tumour bearing mice treated with sunitinib

**Fig. 9:**
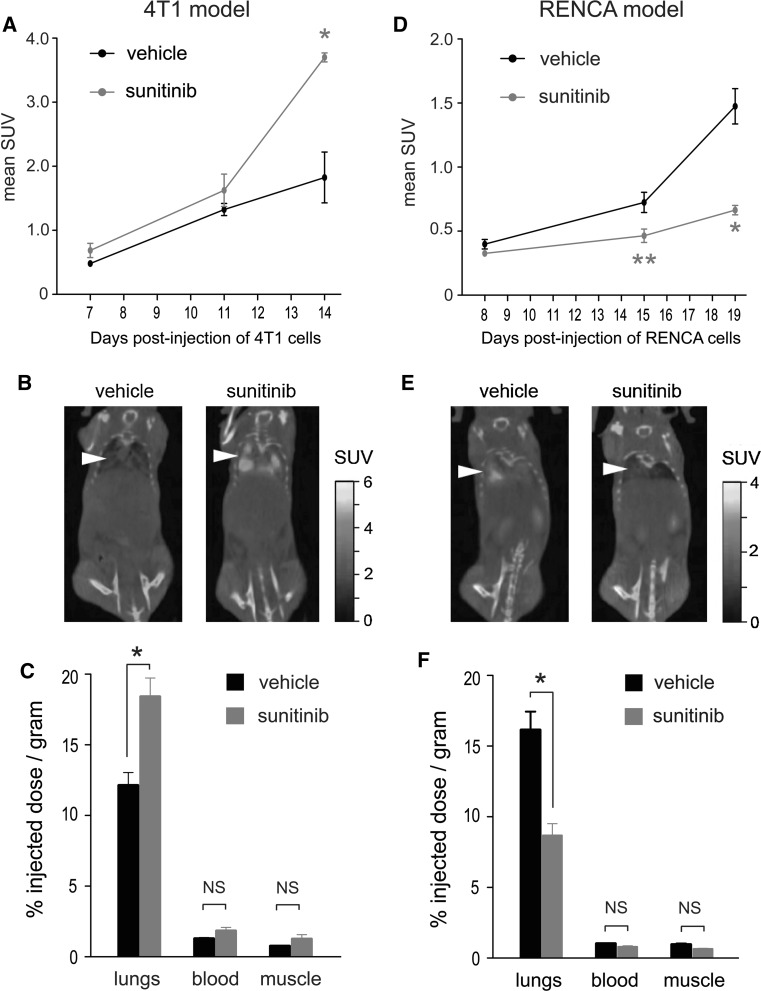
Measurement of tumour glucose uptake. Balb/c mice were injected intravenously with 4T1-luc cells (**a**–**c**) or RENCA-luc cells (**d**–**f**) and then treated with daily vehicle or 60 mg/kg/day sunitinib on a continuous dosing schedule. ^18^FDG uptake was measured by ^18^FDG-PET/CT imaging of live animals or by gamma counting of resected tissues. **a**, **d** *Graphs* show mean standard uptake value (SUV) measured in the lungs of 4T1 (**a**) or RENCA (**d**) tumour-bearing mice using ^18^FDG-PET/CT imaging at the indicated time point. *P = 0.02, **P = 0.002, n = 2 mice per experimental group (left and right lungs from each mouse were analysed separately). **b**, **e** Representative ^18^FDG-PET/CT images are shown of 4T1 tumour bearing mice at 14 days (**b**) and RENCA tumour bearing mice at 19 days (**e**) after tumour cell implantation, respectively. *Arrowhead* indicates location of chest cavity. **c**, **f** Gamma counting was used to measure the amount of ^18^FDG present in lungs, blood and muscle obtained post-mortem from 4T1 (**c**) or RENCA (**f**) tumour bearing mice at 14 days (**c**) or 19 days (**f**) after tumour cell implantation, respectively. Counts were corrected to the tissue mass in order to calculate the percentage injected dose present per gram of tissue. The *graphs* show the percentage injected dose per gram of tissue ± SEM. *P = 0.01, n = 7 − 10 mice per experimental group. NS = no significant difference

Markers associated with increased hypoxia and increased glucose metabolism were both elevated in 4T1 lung tumour nodules in sunitinib treated mice (see Fig. 7). We therefore addressed whether a functional increase in glucose metabolism was also observed in these 4T1 tumours. Balb/c mice were injected intravenously with 4T1-luc or RENCA-luc tumour cells and then commenced continuous treatment with vehicle or 60 mg/kg/day sunitinib. Glucose metabolism was assessed using both in vivo ^18^FDG-PET/CT imaging and ex vivo quantification of ^18^FDG uptake. The presence of multiple tumour nodules in the lungs of the mice prevented us from assessing ^18^FDG uptake within individual lesions. Instead, tumour glucose uptake was quantified in a semi-quantitative fashion by measuring glucose uptake in the entire lung. In the 4T1 model, the uptake of ^18^FDG in the lungs of sunitinib treated mice was significantly increased compared to vehicle treated mice at 14 days after injection of tumour cells (Fig. 9a–c). In contrast, the uptake of ^18^FDG was significantly suppressed in the lungs of RENCA tumour bearing mice treated with sunitinib compared to vehicle controls (Fig. 9d–f). Since no significant difference in tumour burden between vehicle and sunitinib treated mice is observed in the 4T1 model (for example see Fig. 5a–d), the difference in ^18^FDG uptake measured is most likely due to increased glucose metabolism occurring in the 4T1 lung tumour nodules of sunitinib treated mice.

## Discussion

Therapeutic targeting of the VEGF signalling pathway can suppress tumour growth by blocking tumour angiogenesis [20, 25, 28]. For example, the potent VEGF receptor tyrosine kinase inhibitor sunitinib can extend both progression free survival and overall survival in mRCC patients [44, 45]. However, the clinical efficacy of VEGF pathway targeted agents is hindered by the existence of both intrinsic and acquired tumour resistance [7, 50]. Alarmingly, recent pre-clinical studies have shown that VEGF receptor inhibitors, including sunitinib, may paradoxically promote the growth of metastases in mice [13, 18, 47, 52]. In the present study we show that the ability of sunitinib to promote the growth of metastases is both tumour cell line dependent and sunitinib dose dependent. We also confirm that metastatic disease in mice can be resistant to sunitinib and show that this resistance is associated with a poor outcome. However, in parallel we also show that sunitinib can suppress the growth of metastatic disease in mice and that this is associated with an overall survival benefit.

First we examined the ability of sunitinib to mediate a “conditioning effect” which promotes the seeding and growth of tumours at the site of metastasis. This was based on a previous study, in which treatment with sunitinib prior to intravenous injection of tumour cells was shown to promote metastasis [18]. In our study we demonstrate that not all tumour cell lines may be susceptible to this conditioning effect, since prior treatment with sunitinib only resulted in enhanced seeding of metastasis in mice inoculated with 4T1 cells, but not RENCA cells. In addition, we found that the conditioning effect was only observed when mice were administered doses of sunitinib that produce sustained micromolar circulating concentrations of drug. In patients, circulating concentrations of sunitinib do not typically exceed 200 nM [9, 24]. Moreover, a meta-analysis of pharmacokinetic data demonstrates that, within the clinically relevant dose exposure range, increased circulating levels of sunitinib are in fact associated with improved clinical outcome in mRCC patients, including longer time to progression and longer overall survival [31]. Sunitinib is a relatively selective receptor tyrosine kinase inhibitor at nanomolar concentrations [1, 41, 46], but at micromolar doses it is likely to exert many off-target effects. Consequently, the observation that high dose sunitinib pre-treatment of mice can promote tumour metastasis may be of little relevance in patients.

Whilst this manuscript was under revision, another study [11] reported that sunitinib only promotes metastasis when administered to mice at high doses, which supports the findings reported here. Chung et al. suggested that micromolar doses of sunitinib can promote tumour cell extravasation in the lung by compromising endothelial cell barrier integrity. However, we did not observe enhanced extravasation of tumour cells in the lungs of mice treated with high doses of sunitinib. Instead, we found that tumour cells remained trapped as emboli in the lung vasculature, even at 24 h post-injection, and that the frequency of these emboli was increased in mice pre-treated with high doses of sunitinib. Interestingly, we found that 4T1 tumour cell emboli were trapped preferentially in areas of the lung with low pericyte coverage. Since we also show that high doses of sunitinib lead to reduced pericyte coverage in the lung vasculature, it is possible that sunitinib enhances 4T1 lung metastasis due to the propensity for 4T1 emboli to preferentially seed in vessels with low pericyte coverage. It should be noted that pre-treatment of mice with lower sunitinib doses did not reduce pericyte coverage in the lung. We conclude from this that the pericyte depletion effect noted with 120 mg/kg/day sunitinib is likely an off-target effect of the drug. Of interest, previous studies have demonstrated that pericytes in the primary tumour can limit metastasis [13, 64]. However, we believe this is the first demonstration that pericytes may also limit the seeding of cells at the site of metastasis. Importantly, pericytes can signal in a paracrine fashion to endothelial cells, and may also control capillary constriction, which could both potentially be involved in regulating the metastatic niche [4].

We also examined the effects of sunitinib when it was administered to mice after the intravenous inoculation of tumour cells. While sunitinib failed to suppress the growth of 4T1 lung metastases in this scenario, the same drug suppressed the growth of RENCA lung metastases. Investigating the underlying mechanisms for this differential response, we found that whilst the vascularisation of RENCA tumours was potently inhibited by sunitinib, 4T1 tumour nodules continued to grow with a well-vascularised viable tumour rim. Functional imaging performed in mRCC patients demonstrates that sunitinib refractory lesions often show signs of extensive central devascularisation, but that the rim of these tumours continues to be well vascularised [56, 57]. Therefore, continued vascularisation in this viable tumour rim may be a distinguishing feature of some lesions that are refractory to angiogenesis inhibition. The vascularisation mechanisms in this viable rim deserve further study, as they may present a target for therapeutic intervention. Notably, we observed that 4T1 tumours recruited more pro-angiogenic Gr1+CD11b+ myeloid cells than RENCA tumours and that these cells were associated with the vasculature in 4T1 tumours, but not in RENCA tumours. These myeloid cells mediate resistance to angiogenesis inhibitors in several pre-clinical models, including subcutaneously implanted 4T1 tumours [26, 37, 49, 54]. The recruitment of these cells may therefore contribute to the ability of 4T1 tumours to remain vascularised and progress, despite sunitinib treatment. Other possible explanations for persistent growth of tumours despite sunitinib treatment are: the presence of alternative soluble pro-angiogenic factors [34, 61], alternative modes of tumour vascularisation [16, 38] or a hypoxia-driven increase in the stem cell component of tumours [12].

We demonstrated that sunitinib fails to prolong overall survival in the 4T1 model when it was administered after intravenous tumour cell injection, indeed we even observed shortening of overall survival in this scenario. This is in agreement with a previous report, which showed that sunitinib administered after intravenous injection of MDA-MB-231 cells can shorten overall survival in mice [18]. However, in contrast to Ebos et al., we did not find evidence for enhanced tumour growth in mice administered sunitinib after intravenous tumour cell injection, either in the lungs or in other organs (Fig. 5 and data not shown), suggesting that enhanced tumour growth may not account for the premature mortality observed in our study. Interestingly, we observed rapid weight loss in 4T1 tumour-bearing mice treated with sunitinib in comparison to the other experimental groups (see Table 2) suggesting that cancer-associated weight loss (cachexia) in this cohort may be accelerated. The mechanisms driving cachexia in tumour bearing individuals are poorly understood. However, it has been proposed that a major cause of cachexia is the aggressive metabolism of glucose and glutamine by tumours, because this can place an enormous metabolic stress on the body [15, 58]. Importantly, we observed increased tumour glucose metabolism in the lungs of 4T1 tumour bearing mice after 14 days of sunitinib treatment, which coincided with the manifestation of weight loss in these mice. We speculate that this enhanced tumour glucose consumption places a significant metabolic burden on these mice, which could accelerate cachexia. However, we cannot rule out the possibility that other mechanisms, including drug toxicity, may underlie the premature death of 4T1 tumour bearing mice treated with sunitinib. Of interest, we recently examined FDG-PET/CT as a predictive biomarker of response to sunitinib in mRCC. Patients with highly metabolically active disease at baseline responded poorly to treatment with sunitinib in terms of overall survival benefit, in comparison to sunitinib treated patients with less metabolically active disease who survived significantly longer on treatment [36]. Taken together, the pre-clinical and clinical data suggest that a high rate of tumour metabolism may be associated with a lack of response to angiogenesis inhibitors. Moreover, targeting pathways involved in metabolic adaptation to anti-angiogenic therapy might be a potential therapeutic strategy to improve the clinical efficacy of angiogenesis inhibition [40].

**Table 2:** Time taken for 10 % weight loss to occur in each experimental group

	Treatment received	
Cohort	Vehicle	60 mg/kg/day sunitinib
4T1	20.25 ± 0.86 days	16.25 ± 0.66 days**
RENCA	23.25 ± 0.29 days	35.25 ± 0.60 days
Tumour free	No weight loss observed	37.00 ± 0.82 days

Mice were inoculated intravenously with 4T1-luc or RENCA-luc tumour cells, or left tumour free, and were then treated continuously with daily vehicle or daily 60 mg/kg/day sunitinib and weight loss was recorded. The time taken for 10 % weight loss to occur in each experimental group ± SEM is shown. n = 8 mice per experimental group

** *P* = 0.004 when compared to 4T1 tumour bearing mice treated with vehicle

The experimental models used here have their limitations. In particular, our study was limited to the use of only two murine tumour cell lines and only one angiogenesis inhibitor, so extrapolation to human subjects and other drugs that target the VEGF pathway should be approached with due caution. In addition, our study did not address the ability of sunitinib to promote metastasis from the primary tumour, so no inference can be made regarding the effects of sunitinib on the formation of distant metastasis when it is used in the neoadjuvant setting. Interestingly, recent work suggests that although tyrosine kinase inhibitors can promote metastasis in some mouse models, anti-VEGF monoclonal antibodies do not [13, 55]. In spite of these limitations, our study does have important implications. We did not observe a conditioning effect that promotes the growth of metastases when sunitinib was used at clinically relevant doses, suggesting that a conditioning effect is unlikely to accelerate the growth of metastases in patients. This is important, given that large adjuvant studies of VEGF-targeted agents, such as sunitinib, are currently underway and because patients with metastatic disease also continue to be treated with these agents. Instead, we replicate in mice the responses that are seen clinically with VEGF receptor targeted agents: lack of tumour response to therapy is associated with a poor outcome, whereas suppression of tumour growth is associated with an extension in overall survival [30]. We propose that a mixture of both tumour autonomous mechanisms and elements within the microenvironment conspire to determine the response to anti-angiogenic therapy within individual tumours. In light of these findings, research should now be focused on understanding the aspects of tumour cell biology that determine response and resistance to anti-angiogenic therapies.
